# Dectin-1 Signaling Update: New Perspectives for Trained Immunity

**DOI:** 10.3389/fimmu.2022.812148

**Published:** 2022-02-14

**Authors:** Pablo Mata-Martínez, Marta Bergón-Gutiérrez, Carlos del Fresno

**Affiliations:** Immune response and Immunomodulation Group, Hospital La Paz Institute for Health Research (IdiPAZ), Madrid, Spain

**Keywords:** dectin-1, innate immunity, *Candida albicans*, trained immunity, signaling

## Abstract

The C-type lectin receptor Dectin-1 was originally described as the β-glucan receptor expressed in myeloid cells, with crucial functions in antifungal responses. However, over time, different ligands both of microbial-derived and endogenous origin have been shown to be recognized by Dectin-1. The outcomes of this recognition are diverse, including pro-inflammatory responses such as cytokine production, reactive oxygen species generation and phagocytosis. Nonetheless, tolerant responses have been also attributed to Dectin-1, depending on the specific ligand engaged. Dectin-1 recognition of their ligands triggers a plethora of downstream signaling pathways, with complex interrelationships. These signaling routes can be modulated by diverse factors such as phosphatases or tetraspanins, resulting either in pro-inflammatory or regulatory responses. Since its first depiction, Dectin-1 has recently gained a renewed attention due to its role in the induction of trained immunity. This process of long-term memory of innate immune cells can be triggered by β-glucans, and Dectin-1 is crucial for its initiation. The main signaling pathways involved in this process have been described, although the understanding of the above-mentioned complexity in the β-glucan-induced trained immunity is still scarce. In here, we have reviewed and updated all these factors related to the biology of Dectin-1, highlighting the gaps that deserve further research. We believe on the relevance to fully understand how this receptor works, and therefore, how we could harness it in different pathological conditions as diverse as fungal infections, autoimmunity, or cancer.

## Introduction

The vertebrate immune system is characterized by both an early innate and an adaptive immunity. To activate innate responses, cells of the innate immune system are armed with several receptors called Pattern Recognition Receptors (PRRs), which sense evolutionarily conserved structures. If these structures are displayed by exogenous invading agents, they are called Pathogen Associated Molecular Patterns (PAMPs), and their recognition by PRRs alerts about the presence of an infection (1). Notably, PRRs can also recognize endogenous molecules that are confined at the intracellular level in the steady-state, but that are released or exposed in the membrane of cells when they are damaged or dying; these structures are called Damage-Associated Molecular Patterns (DAMPs) (2). The recognition of either PAMPs or DAMPs by PRRs triggers a number of molecular signaling pathways leading to cell activation, and the generation of diverse responses such as phagocytosis or the release of inflammatory mediators (3).

PRRs can be found as cell membrane-anchored, cytosolic or soluble proteins and are broadly grouped into four families, namely, C-type Lectin Receptors (CLRs), Toll-Like Receptors (TLRs), Nucleotide binding Oligomerization Domain containing (NOD)-like receptors (NLRs), and Rig1-Like Receptors (RLRs) (4).

CLRs comprise more than a thousand of receptors that can be classified in 17 groups (5). In a general and simplified structural view, CLRs consist of an extracellular C-type lectin-like Carbohydrate Recognition Domain (CRD) that binds sugars both in a Ca^2+^-dependent or -independent manner. This is followed by a short transmembrane stalk of variable length prior to the intracellular domain (5), that contains cysteine residues involved in homo- or heterodimerization. This stalk domain lets link the CRD with the transmembrane region which is followed by the intracellular domain. The later harbors signaling motifs such as ITAM (Immunoreceptor Tyrosine-based Activation Motif), ITIM (Immunoreceptor Tyrosine-based Inhibitory Motif), or hemITAM; other CLRs lacking any of these motifs can also trigger intracellular signaling (6, 7).

ITAM-bearing receptors are involved in activation pathways leading to pro-inflammatory responses such as cytokines or Reactive Oxygen Species (ROS) production, and phagocytosis, while ITIM-bearing CLRs dampen such inflammatory reactions triggered by heterologous receptors in *trans* (7). For ITAM-coupled CLRs, two phosphorylated tyrosines are required to achieve a full downstream activation, which are usually provided by two tyrosine residues present in the ITAM motifs (8). However, some of these receptors only bear a single tyrosine in their intracellular fraction, defining a hemITAM-containing CLR (5). Thus, these receptors activate downstream activating pathways through dimerization, although this mechanism has only been formally demonstrated for CLEC-2 (9, 10).

In here, we aim to compile and update the current knowledge on the CLR DEndritic cell-associated C-type lecTIN receptor-1, Dectin-1, its expression pattern, ligands, and the signaling pathways ignited downstream its activation. Of note, this receptor is a key component of the revolutionizing concept of trained immunity (TI), a process of long-term memory developed by innate immune cells. We will explore the signaling pathways involved in TI downstream Dectin-1, and how the updated view of these molecular routes can contribute to a better understanding of the training process.

## Dectin-1, the “Master” β-Glucan Receptor

Dectin-1, also known as β-glucan receptor, is a type II transmembrane lectin belonging to the family of CLRs (11). Structurally, Dectin-1 has a single CRD that specifically recognizes polysaccharides defined as β- (1 → 3)/(1 → 6) – glucans, initially described to be present in the cell wall of certain pathogens including fungi and some bacteria (12, 13). Interestingly, Dectin-1 lacks the Ca^2+^-binding site in its CRD, therefore, ligand recognition is Ca^2+^-independent (14). Furthermore, its intracellular cytoplasmic tail contains an hemITAM signaling motif (15). Adachi et al. suggested that for the specific ligand-Dectin-1 receptor interaction, two conserved amino acid residues (Trp 221 and His 223) are necessary, since they are important for the formation of ligand binding sites and contribute to receptor functionality (15).

Encoded by the *CLEC7A* gene (11), Dectin-1 is widely expressed in the myeloid lineage, which involves macrophages/monocytes, Dendritic Cells (DCs), and neutrophils, as well as in γδ T cells from the lymphoid lineage (13, 16–19). According to its expression pattern at Immgen (20), the highest expression of this receptor in mice can be found in neutrophils, macrophages, and monocytes while DCs express lower levels. This wide expression pattern across the immune system suggests a highly complex variety of responses, and subsequent signaling following Dectin-1 activation. An example of this diversity and versatility is provided by human DCs.

DCs can be classified in humans, at least, in two different cellular subsets based on their function and specific markers, namely myeloid CD11c^+^CD123^-^ (mDCs) and CD11c^-^CD123^+^ plasmacytoid DCs (pDCs) (21). Both subsets express Dectin-1, although much higher levels are present in mDCs (22, 23). Interestingly, Dectin-1-activated pDCs promote Th2-type T cell responses, while Dectin-1-activated mDCs suppress Th2 responses. This differential response relies on a specific OX40L expression pattern triggered following Dectin-1 engagement in pDCs and mDCs (22).

Despite this well-established expression pattern in immune cells, some studies have shown Dectin-1 to be expressed by non-immune cells located in epithelial tissues (24). Epithelial tissues are composed of many different cells such as epithelial cells, melanocytes, keratinocytes, and immune cells such as Langerhans cells. Among these cell subsets, Langerhans cells express Dectin-1, but also keratinocytes are Dectin-1^+^ cells (25). The treatment of keratinocytes with pro-inflammatory cytokines such as interferon-γ (IFN-γ), interleukin (IL)-1α, IL-6, Tumor Necrosis Factor-α (TNF-α) or IL-17 leads to increased Dectin-1 expression, while IL-4 and IL-13 do not induce any change over Dectin-1 levels (25). This would imply that pro-inflammatory environments favor the expression of PRRs in non-immune cells, with functional consequences. In this sense, expression of Dectin-1 in human corneal epithelial cells has been demonstrated, as well as in rat models during *Aspergillus fumigatus* infection (26–28). Furthermore, Dectin-1 expression have been also detected in primary human intestinal epithelial cells from ileum and colon as well as cell lines, conferring the capacity to produce pro-inflammatory cytokines in response to β-glucans (29). Similar results were obtained in human bronchial epithelial cells, which generated a pro-inflammatory response against *Haemophilus influenzae* in a Dectin-1-dependent manner (30). Taken altogether, it could be suggested that Dectin-1 plays a critical role against invading pathogens in mucosal surfaces, involving other cell types than myeloid subsets. However, deeper research is needed to achieve a complete understanding of the regulation, outcomes, and clinical relevance of Dectin-1 expression in non-myeloid cells.

## Dectin-1 Ligands

Dectin-1 was firstly described as the main β-glucan receptor (31). Nonetheless, some other Dectin-1 ligands have been depicted, which could lead us to propose Dectin-1 as one of the most versatile receptors in myeloid cells, considering also the variety of processes in which it is involved such as allergy, cancer, autoimmune disease, sterile inflammation, and even ageing (13). In this section we will summarize the main exogenous and endogenous Dectin-1 ligands described to date, focusing on its pathophysiological relevance and the immune response underneath ligand-receptor recognition (**Table 1**).

**Table 1:** Dectin-1 ligands described up to date.

Ligand	Structure	Pathology	Recognized by	Physiological relevance	References
**β-glucans**	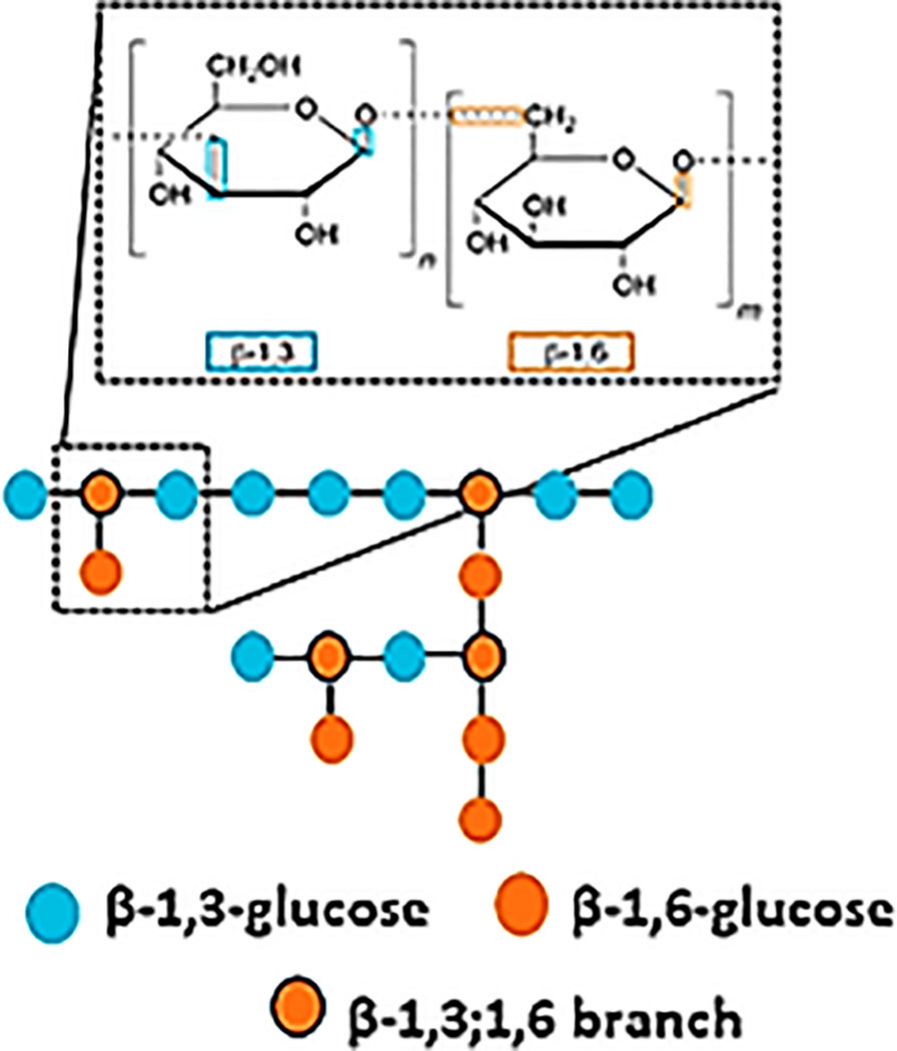	Microbial infections	Neutrophils	Proinflammatory: IL-1β, IL-6, IL-23, TNF-α and ROS production.	(20, 24–28)
			Macrophages	Phagocytosis of pathogens.	
			Monocytes		
			Dendritic cells		
			Keratinocytes		
			Epithelial cells		
**Galectin-9**	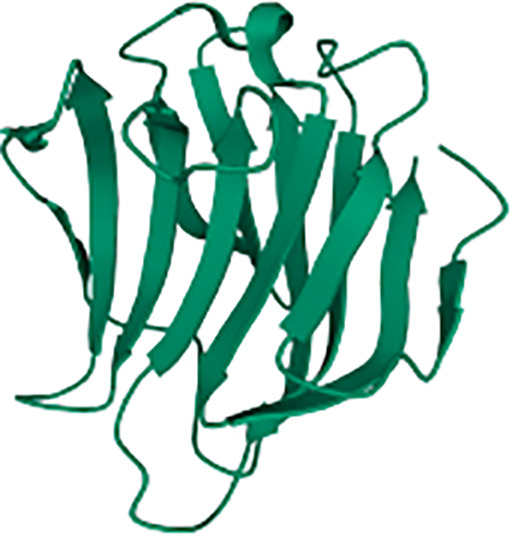	Autoimmune diseases	Neutrophils	Anti-inflammatory: tolerance and induction of oncostatin M overexpression.	(32)
			Macrophages		
			Microglia		
			Dendritic cells		
		Cancer	Macrophages	Immune tolerance: low MHC-II, iNOS and TNF-α, high CD206 expression.	(33)
				Tumor progression.	
**Annexins**	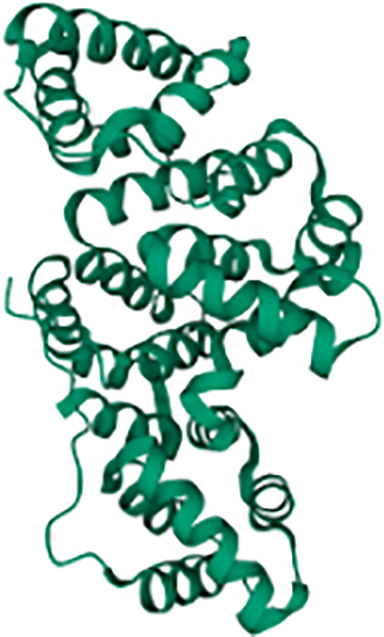	Autoimmune diseases and aging	Dendritic cells	Immune tolerance: reduced ROS production, dampening CD80 and CD86 production.	(34)
**Vimentin**	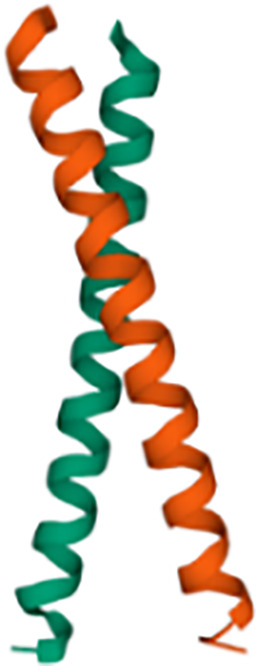	Atherosclerosis	Myeloid cells	Proinflammatory.	(35)
		Ischemia/reperfusion	Macrophages	Proinflammatory.	(36)
			Neutrophils	M1-macrophage recruitment, myocardial injury, and apoptosis.	
		Obesity	Macrophages	Proinflammatory: insulin resistance.	(37)
**Tropomyosin**	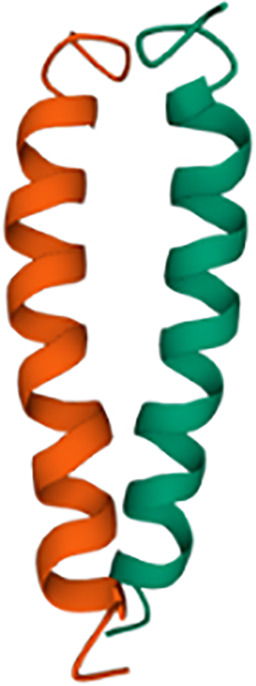	Allergy	Epithelial cells	Immune tolerance: reduced allergic symptoms and IL-33 dampening.	(38)
**N-glycan**	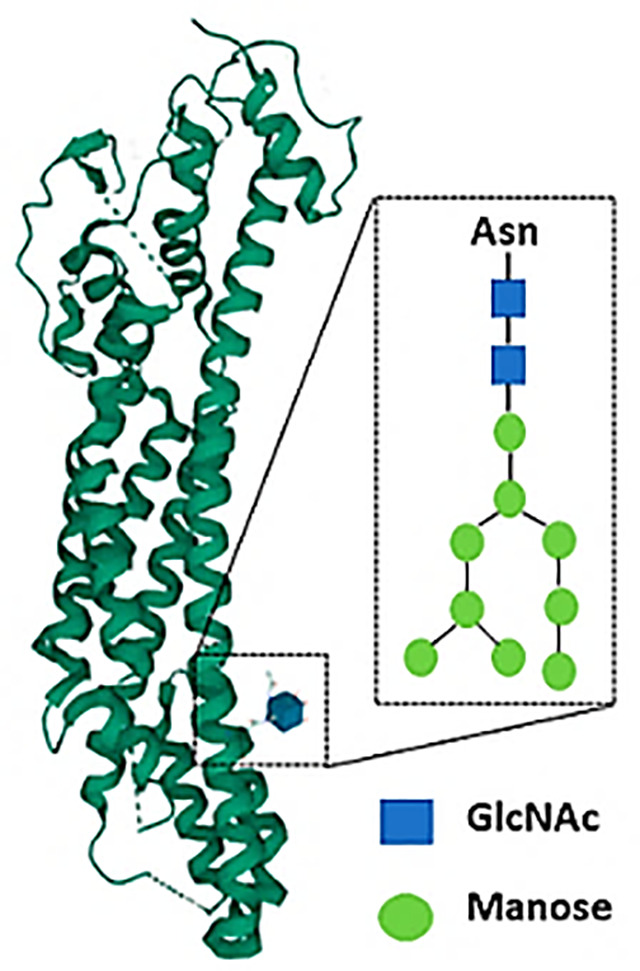	Cancer	Dendritic cells	Proinflammatory: Anti-tumor response *via* induction of natural killer cells cytolytic capacity.	(39, 40)
			Macrophages		

### Microbial PAMPs

Despite the well-established fact that β-1,3-glucans with β-1,6 branching constitute the main Dectin-1 ligands, β-glucans constitute a highly heterologous group of polysaccharides, from a structural point of view. Ligand molecular size, polymer length, branching and solubility are key conditioners of Dectin-1-mediated immune responses. β-glucans for Dectin-1 include curdlan, whole glucan particles (WGP), paramylon, zymosan, and laminarin (41). The first four are large and insoluble β-glucan particles whereas laminarin is soluble. Considering IL-1β production as a readout, curdlan emerges as a better inducer compared to paramylon and zymosan (41). The relevance of the particle size has been also assessed stimulating human DCs with curdlan versus β-glucan microparticles (200μm and 1-5 μm diameter, respectively). The larger β-glucan particle size, the higher IL-1β, IL-6 and IL-23 production was found following stimulation (42). Furthermore, Goodridge et al. demonstrated in an elegant study that particulate insoluble β-glucans such as zymosan and WGP induced a robust immune response including phagocytosis, TNF-α, IL-6 and ROS production in human macrophages. In contrast, large soluble β-glucans were unable to activate Dectin-1 because the formation of a “phagocytic synapse” was required (43). This was consistent with former reports showing that laminarin, a soluble and linear β−glucan, was a high-affinity Dectin-1 ligand that can block the interaction of the receptor with alternative substrates without an active Dectin-1 signaling in bone marrow-derived macrophages (BMMs) and DCs (BMDCs) (44, 45). Therefore, the size and solubility of β-glucans constitute a key molecular checkpoint to predict immunological outcomes following Dectin-1 activation. These diverse Dectin-1-mediated reactions are key in response to β−glucans present on the surface of *Coccidioides* (41), *Saccharomyces* (32), *Candida* (32) and *Aspergillus* (46), allowing an optimal antifungal defense against these potentially pathogenic fungi.

In addition to this protective antifungal role of Dectin-1, the expression of this receptor in the mammalian intestine impacts the composition of the gut microbiome. In fact, the regulation of intestinal microflora by Dectin-1 plays a critical role in intestinal diseases. Thus, changes in the microbiota as consequence of Dectin-1 absence includes an increased colonization of opportunistic pathogenic fungi such as *Candida* and *Trichosporon* in detriment to *Saccharomyces* predominance. A consequence of the abnormal colonization of *Candida tropicalis* and other fungi in Dectin-1-deficient mice during dextran sulfate sodium (DSS)-induced colitis, is the aggravation of colitis symptoms. Interestingly, some *CLEC7A* polymorphisms associate with severity of ulcerative colitis severity in humans (47), indicating the relevance of Dectin-1 in the regulation of gut inflammation.

In addition to these changes in the mycobiome, Dectin-1 deficiency has been associated with acute changes in the proportion of Gram-negative to Gram-positive bacteria (up to two times less in *Clec7a* ^-/-^ compared to WT), with higher proportions of *Lactobacillus murinus* in Dectin-1-deficient mice (48). However, opposite to above-mentioned abnormal fungi colonization, Dectin-1-dependent changes in bacterial microbiota appear to be protective during DSS-induced colitis by promoting the expansion of regulatory T-cells in a *Lactobacillus*-dependent manner (49). The discrepancy between fungal and bacterial modulation exerted by Dectin-1 in the outcome of colonic pathology indicates that more investigation is needed to understand the relevance of this receptor in the regulation of the gut microbiome, although a differential steady-state microbiota between studies could also introduce some divergences.

### Endogenous Ligands - DAMPs

In addition to the widely described role of Dectin-1 in β-glucan recognition of fungal origin and therefore, in antifungal immune responses, this receptor has been implicated in other pathological conditions named before (13). Herein, some different molecules have been proposed as endogenous Dectin-1 ligands with no fungal intervention.

#### Galectin-9

Galectin-9 is a member of the galectin protein family which can interact with immune checkpoint molecules such as T cell Immunoglobulin and Mucin-domain containing-3 (TIM-3), implicated in T cell death, among other functions (33, 50). The capacity of interaction also with Programmed cell Death protein 1 (PD-1) makes galectin-9 a protein with immunomodulatory effects in T cells (34). However, galectin-9 is not only recognized by T cells but also by myeloid cells.

Deerhake E. et al. have demonstrated that Dectin-1 signaling in myeloid cells limits neuroinflammation in an experimental autoimmune encephalomyelitis (EAE) model (38). This immunoprotective role is achieved by the production of oncostatin M in neutrophils, a well-established neuroprotective cytokine (51, 52). Neutrophils appears to be the best oncostatin M producers, as microglia, the main resident immune cell in the central nervous system, monocytes, and BMDCs showed a mild oncostatin M production compared to neutrophil population. In this study, galectin-9 upregulated oncostatin M production in a Dectin-1-dependent manner. Moreover, the treatment with an anti-galectin-9 antibody led to an exacerbated disease in Wild-Type (WT) compared to *Clec7a* ^-/-^ mice, establishing a positive outcome connection in EAE between galectin-9 and Dectin-1. Thus, Dectin-1 signaling is involved in central nervous system sterile inflammation (38).

In cancer, galectin-9 expression is induced. This overexpression may contribute to the establishment of an immunosuppressive tumor microenvironment, and therefore, galectin-9 levels could be associated with poor prognosis. However, other reports have found a positive correlation between galectin-9 expression levels and a better outcome (34, 53, 54). This controversy could be attributed to the variety of processes in which galectin-9 is implicated such as apoptosis, cell adhesion and immune modulation (54). The recognition of galectin-9 by Dectin-1 has been also described in a mouse model of pancreatic carcinoma. Daley D. et al. demonstrated that galectin-9 binds both murine and human Dectin-1, in a dose-dependent manner (55). In this tumoral context, Dectin-1 has been shown to play a critical role in tumor progression supporting immunosuppressive responses. Infiltrating tumor-associated macrophages from *Clec7a* ^-/-^ mice are characterized by higher Major Histocompatibility Complex–II (MHC-II), TNF-α and inducible Nitric Oxid Sintase (iNOS) expression, but lower CD206 compared to macrophages from *Clec7a^+/+^* mice, suggesting a Dectin-1-mediated M2-like reprogramming of myeloid cells in pancreatic carcinoma. Additionally, the blockade of either galectin-9 or Dectin-1 associated with a pro-immunogenic reprogramming (55).

Thus, galectin-9 could be considered as an endogenous Dectin-1 ligand with a strong correlation with immunotolerant phenotypes.

#### Annexins

Annexins are cytosolic proteins associated with cytoskeleton components or membrane proteins that mediate the interaction between the cell and the extracellular matrix. During apoptosis, members of the annexin protein family bind negatively charged phospholipids on the surface of apoptotic cells (35).

Bode K. et al. have reported that annexins A1, A5 and A13 decorating apoptotic cells are recognized with high affinity by Dectin-1 (36). However, these annexins bind to a distinct binding site than β-glucans. Dectin-1 recognition of apoptotic cells triggers a specific phosphorylation pattern of proximal signaling components (Spleen tyrosine Kinase –Syk–, as discussed below), leading to the generation of ROS without cytokine production. Dectin-1 recognition of apoptotic cells generated an immunosuppressive environment to inflammatory stimuli in *trans*, with reduced IL-6 and IL-12 production and a dampened expression of co-stimulatory molecules such as CD80 and CD86 in DCs (36). In line with the immunosuppressive phenotype induced by Dectin-1, *Clec7a^-/-^* mice showed aged-related symptoms of autoimmunity and enhanced immune responses against apoptotic cells-derived antigens (36).

Altogether, this association of annexins and Dectin-1 suggests that the receptor could be relevant in autoimmune and aging-related diseases.

#### Tropomyosin

Tropomyosin is a structural filamentous protein found, among other structures, in actin-based cytoskeletons. Gour N. et al. (37) revealed the role of Dectin-1 in a house dust mite-induced allergy mouse model. They showed that *Clec7a* ^-/-^ mice presented with more allergic symptoms compared to WT. Type II innate lymphoid cells, which participate in allergic disease maintenance (56), are regulated by IL-33, among other cytokines (57). The antibody-blockage of the IL-33 receptor reduced the aberrant mucus production and airway hyperresponsiveness observed in *Clec7a^-/-^* mice, which demonstrated that house dust mite-induced allergy is mediated by IL-33 downstream Dectin-1 (37). Importantly, Gour N. et al. demonstrated that this house dust mite allergy is mediated through the recognition of tropomyosin by Dectin-1 instead of the β−glucans that mites could harbor. Interestingly, this recognition also occurs with shrimp tropomyosin but not with plant tropomyosin obtained from alders and peanuts. This divergency in tropomyosin recognition could be explained because, at the structural and conformational level, tropomyosin of vegetal origin resembles vegetal β-glucans, lacking the β-1,6 branching needed for Dectin-1 recognition (39).

Despite there is no molecular signaling described for tropomyosin/Dectin-1-mediated IL-33 dampening, this axis generates an immunotolerogenic state that avoids aberrant allergic symptoms.

#### Vimentin

Vimentin is a widely expressed intermediate filamentous protein with structural functions. This protein can be also found in the cell surface of viable and apoptotic cells, and even in the culture media, as macrophages secrete vimentin in response to proinflammatory stimulus (40, 58). Thiagarajan P. et al. described the capacity of Dectin-1 to recognize secreted vimentin present in human atherosclerotic plaques. However, no reports detailing the role, intracellular signaling, or physiological implication of this association were found (59).

Interestingly, in a mouse model of myocardial ischemia/reperfusion injury, the recognition of vimentin by Dectin-1 has been revealed as a potential therapeutic target. After reperfusion, Dectin-1 expression levels in cardiac tissue were dramatically increased due to the recruitment of macrophages and, in a minor portion, neutrophils. However, the infiltration of proinflammatory M1 macrophages, cardiomyocyte apoptosis and myocardial injury got highly reduced in *Clec7a^-/-^* mice compared to WT (60). Thus, the blockage of Dectin-1 in ischemia/reperfusion could be an interesting therapeutic approach.

Dectin-1 activation exacerbates obesity and insulin resistance under a high-fat diet in mice lacking MyD88, a proximal adaptor of most TLRs (61). However, deletion of MyD88 in intestinal epithelial cells protects from obesity in high-fat diet fed animals, with improved glucose homeostasis, and reduced hepatic steatosis, fat mass and inflammation, along with an obesity-preventing transferable gut microbiota (62). Interestingly, the Dectin-1-mediated exacerbation phenotype in obesity appears to be mediated by vimentin recognition (61). Thus, the diet-induced upregulation of vimentin in the adipose tissue in obese mice, in combination with Dectin-1 signaling blockade, lead to improved insulin sensitivity and higher macrophage numbers with an anti-inflammatory phenotype. However, as reviewed before, the lack of Dectin-1 alters the gut microbiota also under high-fat diet (61), and this could also have a role in the exacerbation of obesity. These data point to Dectin-1 as a potential therapeutic target of interest also in obesity (61). Therefore, it is tempting to speculate a pro-inflammatory role of signaling pathways triggered by Dectin-1 in metabolic pathologies.

#### N-glycan Structures

Tumor cells usually possess a particular molecular signature based on different post-transcriptional and post-translational aberrant changes. One of these modifications is the expression pattern of tumor glucans (63, 64). Of note, specific tumor-associated glucans have been described as potential Dectin-1 ligands. Based on an experimental metastasis model with the B16 melanoma cell line, Chiba et al. showed a marked enhancement of lung metastasis in Dectin-1-deficient mice. Mechanistically, Dectin-1 binds to N-glycan structures on the surface of tumor cells, that contributes to the eventual cytolytic activation of Natural Killer (NK) cells (65). Interestingly, the increased expression of N-glycans structures in tumor cells has been associated with tumor progression (66), therefore, Dectin-1 activation in myeloid cells could be a relevant anti-tumor response of wide interest in different tumors.

As reviewed in this section, Dectin-1 agonists trigger either pro- or anti-inflammatory responses. These differential reactions are grounded on differential pathways downstream the receptor. In the next section we will review these molecular routes ignited after Dectin-1 engagement.

## Signaling Pathways Downstream Dectin-1

As reviewed before, Dectin-1 activation can lead to numerous downstream cellular responses, including production of cytokines such as TNF-α, IL-1β, IL-2, IL-8, IL-10, IL-12 and CXCL2 (32, 67), induction of phagocytosis (68) and respiratory burst through ROS production (44, 45). In addition to this primary innate events, Dectin-1 signaling orchestrates adaptive immunity, igniting the differentiation of naïve CD4^+^ T cells to a T helper (Th) 1 or Th17 phenotype (69), and the activation of CD8^+^ T cells (70). Therefore, Dectin-1 signaling drives innate immune as well as appropriate adaptive responses, as reviewed elsewhere (71).

**Figure 1:**
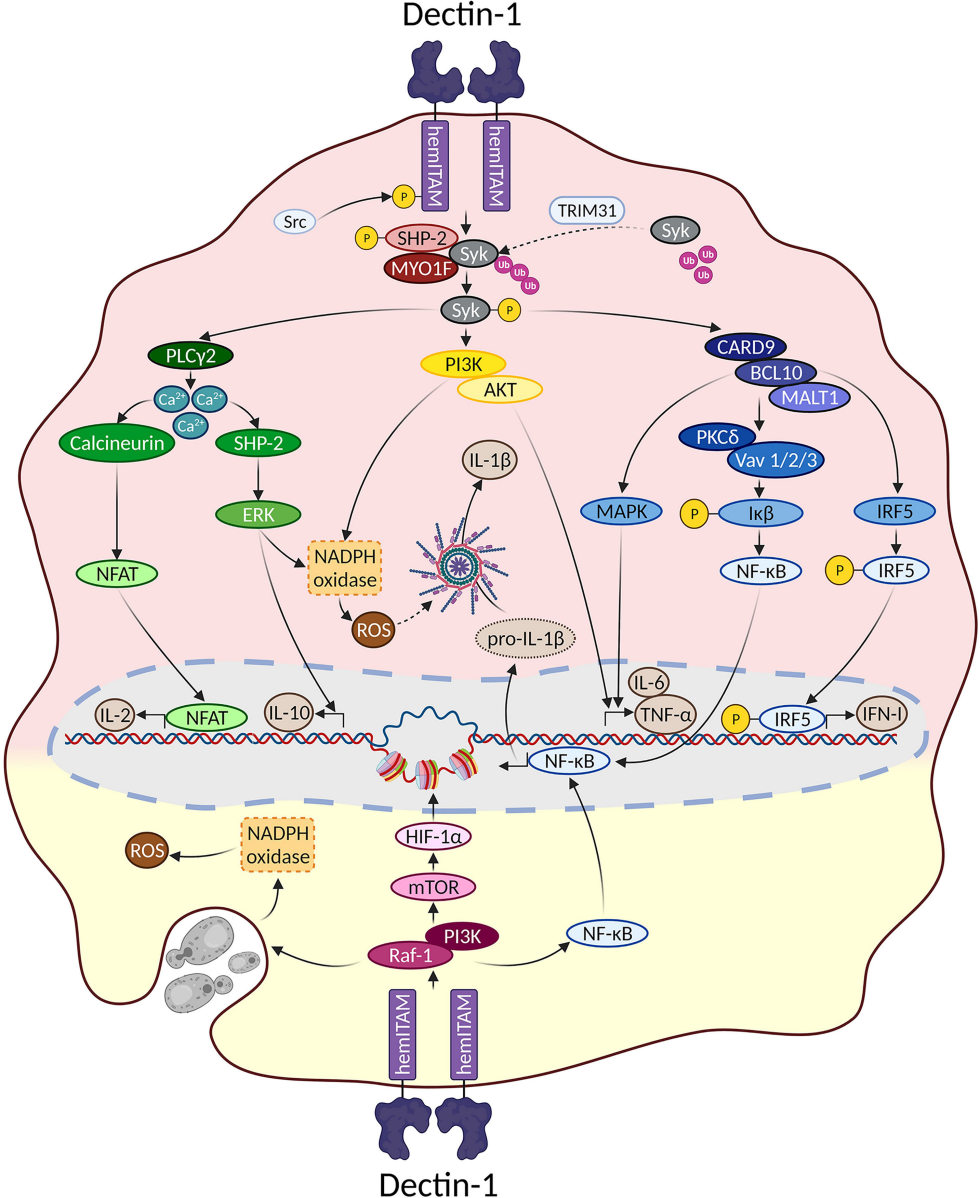
Main signaling pathways downstream Dectin-1. Main signaling pathways triggered downstream Dectin-1 ligation are depicted. In the upper reddish field, Syk-dependent pathways are represented. The lower yellowish field contains the main molecular routes triggered downstream Dectin-1 that do not depend on Syk.

Overall, signaling pathways triggered downstream Dectin-1 could be dissected based on their dependence on the proximal adaptor Syk (**Figure 1**). To draw this picture, it is important to note that most of the experiments performed to address Dectin-1-triggered signaling pathways have been performed in myeloid cells such as monocytes, macrophages, or DCs, both of mouse and human origin. Therefore, although cell type-specific differences should be considered, we assume a “myeloid” signaling pattern all along this review.

### Dectin-1-Triggered Syk-Dependent Molecular Pathways

Syk is a fundamental kinase in the activation pathways triggered by CLRs. Two phosphorylated tyrosines conforming a SH2-docking site are required to achieve a full Syk activation, which are usually provided by the ITAM motif present in the intracellular domains of activated CLRs (8). However, as described before, the intracellular fraction of Dectin-1 only bears a single tyrosine, and therefore Dectin-1 ligation activates Syk through dimerization.

Thus, ligand sensing by Dectin-1 generates a conformational change that triggers an activation signal for the clustering of Dectin-1 hemITAMs at the cytoplasmic tail. Receptor clustering stimulates Src family kinases to phosphorylate the hemITAM motifs in two tyrosine residues, conforming docking sites for Syk kinase (44, 67). Which specific Src kinases among the different members of this family (72) are involved in this activation has not been fully addressed. Nevertheless, pull-down experiments using peptides covering the phosphorylated form of the Dectin-1 intracellular domain, suggested the Hematopoietic Cell Kinase (Hck) and Lck/Yes novel tyrosine kinase (Lyn) as preferential candidates (73). Syk binding to the phosphorylated Dectin-1 hemITAM leads to its own phosphorylation, enzymatic activation and downstream signal transduction (8).

For the above-mentioned molecular events to take place, Syk needs to translocate from its cytoplasmic location to the cell membrane, returning to the cytoplasm following activation. In this sense, the E3 ligase TRIM31 is a crucial factor by interacting with cytoplasmic Syk to catalyze its K27-linked polyubiquitination, that promotes its plasma membrane translocation and binding to the intracellular domain of Dectin-1 (74). After phosphorylation, active Syk moves back to the cytoplasm based on cytoskeleton adaptations controlled by the unconventional myosin MYO1F (75), leading to downstream signal transduction. Notably, the stimulation of Dectin-1 by zymosan induced the phosphorylation of the phosphatase SHP-2. This event was both Src and Syk kinases-dependent (76). *In vitro* experiments placed SHP-2 as a critical factor for Syk activation and subsequent downstream events. Consequently, conditional SHP-2 knockout mice in macrophages and DCs showed enhanced sensitivity to systemic *Candida albicans* infections (76).

Considering the kinetics of all these proximal events, a TRIM31/SHP-2/MYO1F axis could be proposed to achieve a full Syk activation (**Figure 1**), although the understanding of the precise interrelationship between these components needs further studies.

#### Phosphatidylinositol 3-Kinase (PI3K)/AKT

PI3K activation and its downstream effector AKT/PKB is one of the more pleiotropic signaling pathways in nature (77, 78). Ligand recognition by Dectin-1 activates PI3K/AKT in a Syk-dependent manner (79). Surprisingly, the functional contribution of this molecular branch has not been dissected in detail. Still, AKT activation downstream Syk appears to support sustained ROS production (79) once maximal ROS production has been triggered by alternative molecular pathways, as it will be reviewed below.

In conditions where large β-glucan particles cannot be internalized following Dectin-1 engagement, a boosted monocyte activation takes place with enhanced cytokine and ROS production (42). PI3K is crucial for both outputs while Syk only mediates the production of cytokines (80), indicating a role for PI3K in the generation of ROS in a Syk-independent manner (discussed below), but also in the production of cytokines relying on Syk. This Dectin-1/Syk/PI3K pathway involved in cytokine production has been described in response to the fungi *Fusarium proliferatum* (81) and *Blastomyces dermatitidis* (82).

#### Phospholipase C-γ2 (PLC-γ2) and Ca^2+^- Dependent Activation

Syk activation following Dectin-1 engagement leads to increased intracellular Ca^2+^ levels through PLC-γ2 (14), thus triggering Nuclear Factor of Activated T cells (NFAT) activation in DCs and macrophages, and subsequently the expression of Early growth response (Egr) family of transcription factors, cyclooxygenase-2 and IL-2 (45). In addition, the increase in cytoplasmic Ca^2+^ levels leads to the Extracellular Response Kinase (ERK) activation, one of the Mitogen Activated Protein Kinases (MAPK), which drives the production of IL-10 and ROS (83).

Interestingly, functional and biochemical studies suggested that SHP-2 functions downstream Syk after Dectin-1 stimulation, positively contributed to upregulate ROS levels (79). These data point out a different role of SHP-2 respect to the above-mentioned work by Deng et al. (76), although ROS production was not addressed in this latter study. An alternative explanation for these differential results could be that different cell types were used for each study, namely, thioglycolate-elicited peritoneal macrophages (79) versus *ex vivo* differentiated BMMs or BMDCs (76).

#### CARD9/BCL10/MALT1 Complex-Mediated Signaling

Syk activation is required for the formation of a signaling complex composed by CAspase Recruitment Domain 9 (CARD9), a scaffold molecule that binds to B-Cell Lymphoma 10 (BCL10) and forms a trimolecular complex with BCL10 and Mucosa Associated Lymphoid Tissue 1 (MALT1) (84). This complex is essential to trigger Nuclear Factor Kappa-B (NF-κB) that eventually leads to the production of inflammatory cytokines such as pro-IL-1β, IL-6 and TNF-α (84, 85). Of note, Protein Kinase C-δ (PKC-δ) and the Vav family of proteins (Vav1/2/3) are specifically required for NF-κB activation downstream Syk and CARD9 (86, 87). However, these adaptor molecules are dispensable after Syk/CARD9/BCL10/MALT1 signalosome formation for the activation of MAPK, which also contribute to cytokine production (69, 88).

#### Dectin-1/Syk/CARD9/IRF – Type I and III Interferon

A Syk-dependent signaling pathway of particular interest triggered following Dectin-1 engagement leads to the production of type I interferons (IFN-Is). IFN-Is are a family of diverse soluble mediators comprising some barely characterized single members (IFNϵ, IFNτ, IFNκ, IFNω, IFNδ and IFNζ), together with the better studied IFNα (13 in humans and 14 in mouse) and IFNβ (89). A common feature between them all is that they are recognized by a heterodimeric receptor called IFNα receptor (IFNAR), triggering a plethora of IFN-induced genes through a signaling module formed by a Janus Kinase-Signal Transducer and Activator of Transcription (JAK-STAT) axis (90). Since their first description in 1957 (91), IFN-Is are known for their protective effects against viral infections (92). However, further studies demonstrated that IFN-Is are also potent modulators of virtually any immune cell, eventually exerting either protective or detrimental effects in response to different types of infections (93).

Interestingly, several studies have demonstrated that IFN-Is are also potent modulators of immunity during fungal infections. In this sense, pDCs, the main cellular source of IFN-I in the body, were critical against *Aspergillus fumigatus* infection, and IFNAR was fundamental in this effect (94). Also, genome-wide analysis of *Candida albicans*-infected mouse kidneys indicated a relevant influence for IFN signaling trough JAK-STAT (95), suggesting that IFN-Is were produced during systemic *Candida* infection. Indeed, IFN-I pathway was defined as “central for host defense against *Candida albicans*” in human Peripheral Blood Mononuclear Cells (PBMC), even showing that single-nucleotide polymorphisms in members of the IFN-I signaling route associate with susceptibility to systemic candidiasis (96). Functionally, IFN-I recognition after systemic infection with *Candida albicans* is critical for neutrophil recruitment to the kidney (97) where they will clear the infection (98), but also for protective Th1-like immune responses against *Cryptococcus neoformans* (99).

Notably, Dectin-1 recognition of *Candida albicans* or pure agonists such as curdlan induces IFN-Is (100). It is important to note that Dectin-1 ligation in this context seems to have defined particularities, since the dependence of Dectin-1 for IFN-I production is strain (101) or species-specific, as *Candida glabrata*-induced IFN-I production is Dectin-1 independent (102). A plausible explanation for these divergences is that specific components on the surface of different *Candida* strains or species could be relevant for Dectin-1-triggered IFN-I production. Indeed, this fact might also account for the differential roles conferred to Dectin-1 in the systemic response against two different *Candida albicans* strains (103, 104).

Signaling molecules involved in IFN-I generation following Dectin-1 engagement include Syk, CARD9 and Interferon Response Factor 5 (IRF5) (100). As reviewed before, Dectin-1-triggered IRF5 activation has been also described following recognition of glucans present on tumor cells (65). IRFs are cytosolic transcription factors that once activated migrate to the nucleus, where they are recruited to promoters to start transcription of target genes. Different mechanisms have been described to operate in the activation of IRF5 such as phosphorylation and ubiquitination (105); however how IRF5 is activated downstream Dectin-1 is still unknown.

In this sense, two reports indicated that IFN-I production following endosomal TLR engagement requires IRF5 phosphorylation. This phosphorylation is mediated by the sequential action of the upstream kinases Transforming growth factor β-Activated Kinase 1 (TAK1) and Inhibitor of nuclear factor Kappa-B Kinase beta (IKKβ) (106, 107). Considering the common implication of Syk and CARD9 in NF-κB and IRF5 activation downstream Dectin-1, and that TAK1 is activated downstream the CARD9/BCL10 complex (108), we could speculate that IKKβ phosphorylates and consequently activates IRF5 downstream a Dectin-1/Syk/CARD9/BCL10/TAK1 signaling axis. This topic deserves to be studied in detail, as decoding the mechanisms implicated in Dectin-1-triggered IFN-I production would provide new potential tools against the clinically relevant systemic *Candida albicans* infection, with up to 40% of mortality rate in intensive care units services (109).

Related to IFN-Is, IFN-IIIs comprise a family of 4 IFN-λ members (IFN-λ1/IL-29, IFN-λ2/IL28A, IFN-λ3/IL28B, and IFN-λ4). These proteins are recognized by a heterodimeric receptor composed of the class II cytokine receptor subunit IFN-λR1 (also termed IL-28RA) and a second chain, IL-10R2, that also serves as a subunit of the IL-10 receptor for the IL- 10–related cytokine IL-22 (110). As indicated before, Dectin-1 recognition *in vivo* of *Aspergillus fumigatus* induces the production of IFN-Is, which prime optimal expression of IFN-IIIs in lung homogenates (111). These IFN-IIIs act directly on neutrophils activating antifungal responses. Thus, deficiency in IFN-III signaling due to neutrophil-specific deletion of IFN-λR1 succumb to invasive aspergillosis (112). Importantly, exogenous administration of type I and III interferons rescue defective antifungal responses of Dectin-1-deficient mice, overcoming their increased mortality to *Aspergillus fumigatus* infection (111).

The molecular signaling pathways giving rise to IFN-III production downstream Dectin-1 have not been studied in detail. However, *Aspergillus*-infected murine DCs and neutrophils contribute to the recruitment of pDCs to the lung by releasing the CXCL9 and CXCL10 chemokines in a Dectin-1-, CARD9- and type I and III interferon-dependent manner (113). Under this light, we could speculate there is a comparable induction pathway between IFN-I and IFN-III following Dectin-1 engagement. Noteworthy, genetic studies on the genes encoding IFN-IIIs showed that they have binding sites for the transcription factors NF-κB, IRF3, IRF7 and AP-1 in their promoters (114). And these transcription factors together with IRF1 are involved in IFN-III production after viral infections (115), with no definitive information about the involvement of IRF5. Therefore, the Dectin-1-dependent specific signaling pathway leading to the generation of IFN-IIIs deserves further clarification.

### Syk-Independent Molecular Pathways

Following Dectin-1 engagement, there are also signaling pathways triggered in a Syk-independent manner (**Figure 1**). The initiation of this molecular route relies on the Raf-1 kinase (also known as c-Raf) and PI3K/AKT, leading to phagocytosis and the production of cytokines (13, 68). Curiously, the potential involvement of proximal adaptor molecules in this pathway is poorly defined compared to the Syk-dependent route. Based on the seminal work by Deng et al. , Raf-1 phosphorylation depends on SHP-2 in response to Dectin-1 ligands (76). However, the relevance of Myosin IF (MYO1F) in Syk-independent pathways was not explored (75). Considering the differential implication of MYO1F in PI3K/AKT/mammalian Target of Rapamycin (mTOR) activation after IFNγ/LPS stimulation (116), it would be interesting to address the role of this myosin for the Syk-independent molecular pathways triggered downstream Dectin-1, as well as for SHP-2.

Raf-1 kinase activation following Dectin-1 engagement leads to an alternative pathway for NF-κB activation through the NF-κB subunit RelB, modulating cytokine production and the profile of Th differentiation (117). Notably, this pathway might be cell-type specific as it has only been described in human monocyte-derived DCs.

PI3K/AKT activation is also triggered downstream Dectin-1 in a Raf-1-dependent and Syk-independent manner. This molecular pathway is relevant for cytokine production after Dectin-1 recognition of *Histoplasma capsulatum* (118), and for the phagocytosis of zymosan particles (68). Importantly, the axis formed by Dectin-1/Raf-1/PI3K/mTOR/Hypoxia-Inducible Factor (HIF-1α) is critical for the initiation of the trained immunity program initiated by β-glucans (119, 120) through epigenetic remodeling (**Figure 1**), as it will be discussed afterwards.

### Single Lines for Each Signaling Pathway … ROS Production Is Not as Easy

**Figure 2:**
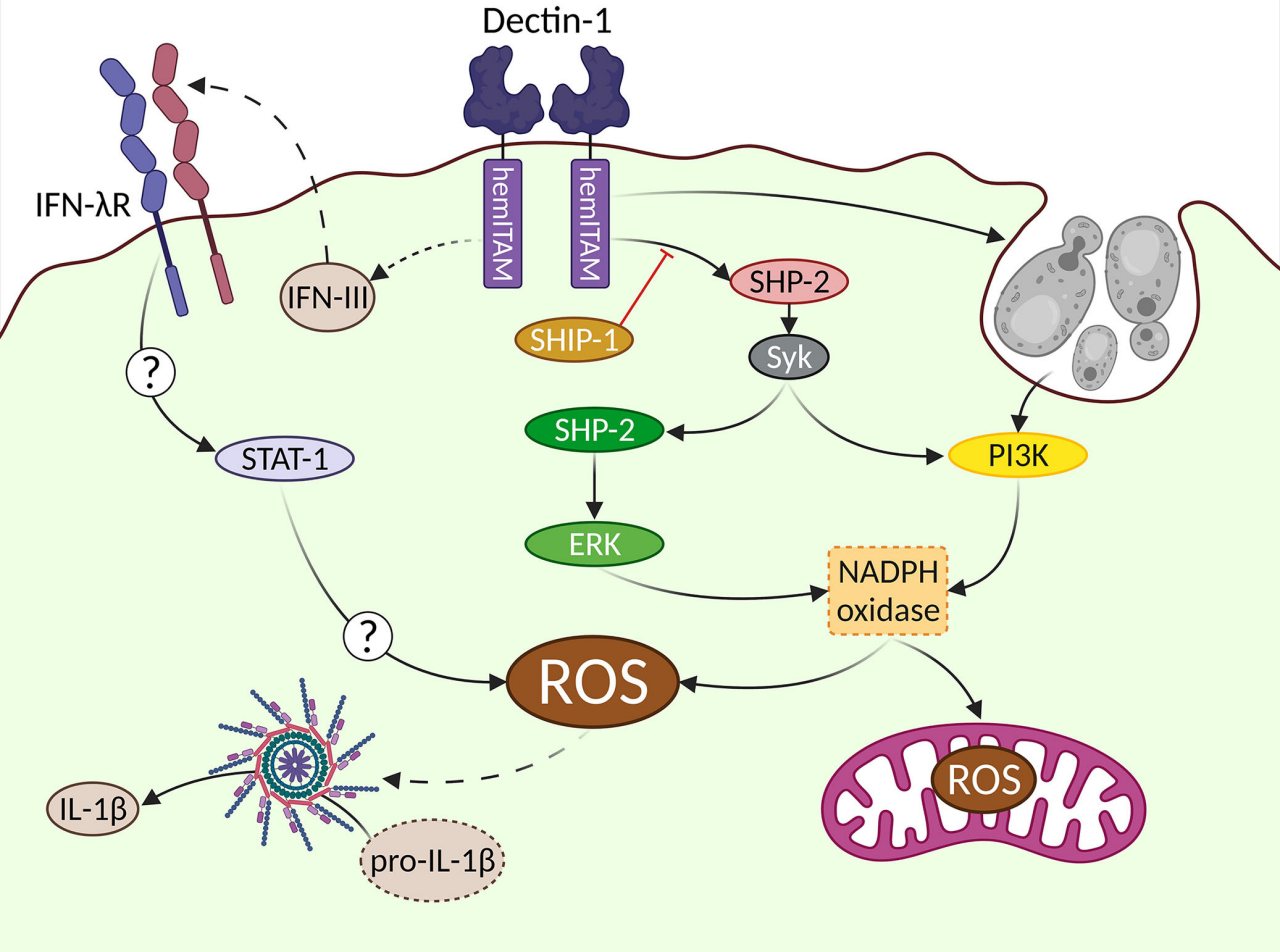
Complex signaling pathways downstream Dectin-1 leading to ROS production. The generation of ROS after Dectin-1 engagement is a relevant event. Different signaling pathways are involved in this process leading to the activation of the NADPH oxidase as a central hub. The uptake of large particulate Dectin-1 ligands triggers a Syk-independent, PI3K-dependent pathway. An alternative Syk/SHP-2/ERK pathway can be also triggered, which can be regulated by the SHIP-1 phosphatase. IFN-IIIs are produced *in vivo* in response to *Aspergillus fumigatus* in a Dectin-1-dependent manner, although their cellular source has not been addressed (represented by dotted lines). These IFN-IIIs are recognized by their specific receptor (IFN-λR), triggering a not fully described STAT1-mediated pathway, that ends up with ROS production. The mitochondrial metabolism also contributes to generate ROS in response to Dectin-1 ligands. In addition to the well-described microbicidal role of ROS, they can act as second messenger signals, exemplified in this figure by the activation of the inflammasome, leading to the processing of pro-IL-1β into bioactive IL-1β.

When addressing molecular pathways, we tend to oversimplify them by connecting different elements of study by means of arrows. However, the actual behavior of these elements is far more complex, with many interrelationships. Due to the complexity of molecular events leading to ROS production, we will try to exemplify these connections on the generation of the oxidative burst downstream Dectin-1, and the eventual production of microbicidal ROS by myeloid cells, mainly neutrophils and macrophages (**Figure 2**). Classically, ROS are conceived as molecules used by phagocytes to destroy pathogens, however, these molecules can also act as a second messenger in different signaling pathways (121).

ROS production following Dectin-1 engagement appears to be strictly Syk-dependent (44, 73, 122). However, under certain circumstances such as inhibition of actin-mediated phagocytosis of large β-glucan particles, ROS production relies on PI3K regardless of Syk (80). ERK was initially proposed as the key downstream effector of ROS production (123, 124). However, different studies demonstrated that ERK activation contributes only partially to the generation of Dectin-1-mediated ROS, accounting for somehow, half of the total generated ROS (73, 83). In addition, it is important to consider the intriguing role of the phosphatase SHP-2 in the Dectin-1-triggered signaling pathway leading to ROS production through ERK activation (79).

Uncovering the complete signaling pathway leading to ROS production is relevant as besides their microbicidal activity, the generation of ROS downstream Dectin-1 is critical for the activation of the NOD-, LRR- and pyrin domain-containing protein 3 (Nlrp3) inflammasome, which is required for the processing of pro-IL-1β into bioactive IL-1β (122). Notably, ERK activity is necessary to produce IL-1β (88, 125). Considering that as indicated before, ERK pathway is only partially relevant for ROS production, alternative ERK-independent signals are required for a complete oxidative burst downstream Dectin-1. In this sense, the PI3K-mediated activation of ROS production under different settings (126), including the sensing of large β-glucans (127, 128), places PI3K as another relevant molecular pathway for the generation of ROS after Dectin-1 engagement.

Eventually, the production of ROS downstream Dectin-1 is fully dependent on the Nicotinamide Adenine Dinucleotide PHosphate (NADPH) oxidase complex (73, 79). This complex is composed of five members (p22, p40, p47, p67 and gp91) that need to be properly assembled to generate ROS (126). The phosphorylation of the NADPH oxidase cytoplasmic members p40 and p47 has been described in response to Dectin-1 agonists as a mechanism involved in the activation of the NADPH complex (129, 130). However, whether Dectin-1-triggered signaling pathways have a direct effect on other members of the complex (131), or the molecular mechanisms implicated in such activation downstream Dectin-1 is still a field of research that deserves much attention.

It is worthy to stress the potential relevance of an alternative source of ROS downstream Dectin-1: the mitochondria (132). Mitochondrial ROS are produced in response to *Aspergillus fumigatus*, with functional relevance in antifungal responses by macrophages. The underlying mechanism relies on the reverse electron transport process (133). Still, the NADPH oxidase is critical for this mitochondrial ROS production, establishing a sequential process of NADPH complex activation before mitochondrial ROS are produced (133).

In any case, the generation of ROS is a powerful tool against invading fungi because the oxidative burst is extremely toxic for these pathogens. However, ROS have pleiotropic effects and consequently their production needs to be tightly regulated in order to avoid harmful effects for the host (134). In this sense, the regulation imposed by phosphatases such as Src Homology 2 (SH2) domain containing Inositol polyphosphate 5-Phosphatase 1 (SHIP-1) appears to be of great relevance. In this sense, the lack of SHIP-1 in BMDCs drives an enhanced ROS production in response to Dectin-1 agonists (73). This SHIP-1-mediated regulatory function supports the active role of PI3K in ROS production downstream Dectin-1, as SHIP-1, along with Phosphatase and TENsin homolog deleted on the chromosome 10 (PTEN), are key regulators of the PI3K enzymatic activity (135).

An extra layer of complexity in the generation of ROS downstream Dectin-1 is the modulation exerted by IFN-IIIs (111). Airway infiltrating neutrophils from mice deficient in IFN-λR1, the specific receptor subunit of IFN-IIIs, showed a great reduction in ROS production following pulmonary *Aspergillus fumigatus* infection (112). Consequently, mice bearing a conditional depletion of IFN-λR1 in neutrophils were extremely sensitive to this infection (112). Therefore, another “line” should be drawn connecting IFN-III production and ROS downstream Dectin-1, with the activation of STAT-1 as the most likely mediator (112, 136). However, the actual mechanisms underlying this connection are not fully explored.

### Modulating Signaling Pathways

CLRs are a wide family of receptors that interact with each other or with other receptor families in a homo- or heterotypic manner, respectively. The latter represents a complex net of interrelationships that goes beyond the scope of this review, and that has been already reviewed elsewhere (7, 137). However, in here, we will address how adjacent and signaling molecules directly modulate Dectin-1-triggered responses. We will focus on phosphatases as classical regulators of signaling cascades, and tetraspanins, interacting proteins with modulatory capabilities.

#### Phosphatases

The Protein Tyrosine Phosphatase Non-receptor type 22 (PTPN22) was known to negatively regulate T-Cell Receptor (TCR) signaling by inhibiting Src kinases and Zap70, the Syk homolog expressed in T cells (138). The relevance of Syk for Dectin-1-triggered responses prompted to investigate the role of this phosphatase downstream Dectin-1. *Ptpn22*-deficient DCs showed enhanced Syk and ERK phosphorylation in response to the Dectin-1 ligand curdlan, giving rise to increased IL-1β production and boosted antigen-specific Th17 responses (139). Comparable enhanced responses were observed in the absence of the membrane-anchored adaptor FcRγ (140). Its association to the SHP-1 and PTEN phosphatases was proposed as the regulatory mechanism, although the role of PTPN22 was not addressed (140). Considering how comparable were the enhanced responses observed in PTPN22- and FcRγ-deficient myeloid cells, it would be interesting to address their potential entwining.

As described before, SHIP-1-deficient BMDCs showed enhanced ROS production in response to Dectin-1 agonists (73). Similarly, the lack of the Sts-1 and Sts-2 phosphatases also led to an specific increased ROS production, with no impact in other readouts such as cytokine production (141). Of note, this enhanced response in the absence of SHIP-1 or Sts phosphatases was exclusively observed in BMDCs but not in neutrophils (73, 141), implying the presence of specific mechanisms in DCs. Furthermore, the precise role of these phosphatases on ROS production is intriguing. As described above, a wide number of readouts downstream Dectin-1 rely on Syk activation (*see* **Figure 1** *, upper area*). However, despite both SHIP-1- and Sts-deficient DCs showed enhanced Syk phosphorylation in response to Dectin-1 agonists, ROS production was the only effector process altered. To explain this fact, we could hypothesize either the existence of alternative regulatory mechanisms downstream Syk that are not involved in ROS production, or the need of phosphatases-insensitive coactivating signals for the rest of effector mechanisms.

As reviewed before, the size of the ligands recognized by Dectin-1 determines the nature of downstream signaling pathways and outcomes. Goodridge et al. (43) described that particulate but not soluble β-glucans induce activating signals due to the formation of phagocytic synapses. During this process, Dectin-1 molecules cluster in a volcano-like structure, excluding the CD45 and CD148 membrane-anchored phosphatases from the initial signalosome. This exclusion permits the activation of Src kinases such as Lyn, giving rise to cytokine and ROS production.

#### Tetraspanins, a Tale of Interactions

Tetraspanins are a family of transmembrane proteins that influence a wide number of biological processes including adhesion, proliferation, antigen presentation or endocytosis, among others. They interact with diverse proteins such as immunoreceptors or integrins to establish functional multimeric complexes denominated tetraspanin-enriched microdomains (142). Some members of this family are known to interact with Dectin-1, modulating the signaling pathways and outcomes triggered following receptor engagement.

Chronologically, CD63 was the first tetraspanin described to interact with Dectin-1 in a screening of this family of proteins in human DCs (143). CD63 was internalized along with Dectin-1 during the phagocytosis of *Saccharomyces cerevisiae* yeasts, although the functional relevance of this interaction was not addressed (143). However, as antibody-mediated crosslinking of CD63 improved the chemokine-induced migration of DCs (143), a co-stimulating role could be speculated for CD63 in Dectin-1-mediated responses.

The interaction between the tetraspanin CD37 and Dectin-1 was described next. In this case, the lack of CD37 in macrophages enhanced IL-6 production in response to Dectin-1 agonists such as zymosan and curdlan, despite a reduced cell surface expression of the CLR (144). Interestingly, the phagocytosis of zymosan was not affected in CD37-deficient cells, suggesting a function-specific role of this tetraspanin that could be attributed to its participation in the Syk-dependent pathway downstream Dectin-1 (*see* **Figure 1** *, upper area*). Furthermore, serum levels of *Candida*- or Zymosan-specific IgA were enhanced in CD37-deficient mice after the systemic administration of both agents (145). In line with these results, mice lacking CD37 were highly resistant to a lethal systemic *Candida albicans* infection. Altogether, the tetraspanin CD37 appears to have a regulatory role on Dectin-1 signaling.

The next tetraspanin member described to be an interaction partner of Dectin-1 was CD82. In this case, CD82-deficient macrophages showed reduced Src and Syk phosphorylation in response to *Candida albicans*, leading to a decreased cytokine and ROS production. Consequently, mice lacking CD82 were more susceptible to systemic candidiasis (146), suggesting that this tetraspanin is required for optimal Dectin-1-mediated responses. A similar function was attributed to the Ms4a4a tetraspanin, as its deficiency also dampened Syk phosphorylation and the production of cytokines and ROS (147). Interestingly, under the experimental lung metastasis model induced by systemic administration of B16F1 melanoma cells, *Ms4a4a*-deficient mice showed a larger number of metastatic nodules than WT animals. In parallel experiments, *Clec7a^-/-^* mice also showed higher numbers of metastases (147). These results suggest that the tetraspanin Ms4a4a is required for the antitumor effect triggered after Dectin-1 recognition of tumor cells-expressed glucans (65).

Altogether, these data point to tetraspanins as target candidates in clinicopathological conditions where the modulation of Dectin-1-triggered responses could be of potential interest.

## Trained Immunity

When innate immune cells are challenged with certain stimuli, they can develop long-lasting effects that result in an enhanced pro-inflammatory response to a second challenge; this process is referred to as trained immunity (TI) (119). An important feature of this memory is its heterologous capacity of recalling, as the boosted inflammatory response can be revealed by unrelated stimuli to the ones that induced the training process (1). For instance, the triggering of TI in myeloid cells with stimuli of fungal origin confers protection against bacterial or viral infections. Mechanistically, a shift towards glycolytic metabolism and the epigenetic reprogramming of promoters of inflammatory mediators drives the characteristic long-lasting inflammatory response of TI. These epigenetic changes are mainly mediated by histone modifications such as methylation or acetylation (119, 148–150), resulting in an open-chromatin condition. This generates a pre-activated “stand-by” transcriptional status that enables an enhanced proinflammatory response after a second stimulus.

In addition to this cell-autonomous mechanisms, it is known that numbers of hematopoietic progenitors are increased after exposition to TI inducers. Those progenitors also experience transcriptomic and metabolic changes that contribute to maintain durable memory of newly generated innate immune cells upon a second stimulus (151, 152). Importantly, TI takes place in mice lacking adaptive immunity such as Severe Combined ImmunoDeficiency (SCID) (153) or *Rag1*-deficient (119), inducing protecting responses against lethal *Staphylococcus aureus* infection and systemic candidiasis (119, 153–155). Taking these data altogether, TI is considered a *de facto* innate immune memory (1).

Among the best characterized TI inducers, we can enumerate the tuberculosis vaccine Bacillus Calmette Guerin (BCG) and β-glucans of fungal origin. Although NOD2 has been the main receptor implicated in the induction of TI by BCG (153), Dectin-1, as the prototypical receptor of β-glucans, emerges as a critical sensor involved in the induction of TI, but also as a collaborative receptor for the recognition of BCG (156).

### Dectin-1-Triggered Signaling Pathways Leading to Trained Immunity

From the first mechanistic description of TI in myeloid cells, the Dectin-1/AKT/mTOR/HIF-1α axis arose as a critical signaling pathway (157). This notion was grounded on an enhanced survival of mice infected with *Staphylococcus aureus* previously exposed to β-glucan, depending on the expression of HIF-1α in the myeloid compartment (157). Notably, for the induction of TI, the mTOR/HIF-1α axis acts at two levels: i) driving the metabolic shift towards a glycolytic phenotype with consequences on the epigenetic status of the cell, ii) upregulating the expression of glycolysis related genes (157).

During the establishment of the Dectin-1-mediated trained process, a massive metabolic reprogramming takes place. Glycolysis, glutaminolysis and cholesterol synthesis pathways get activated and become indispensable for the TI induction by β-glucan (158). The increase in glycolysis is accompanied by an accumulation of tricarboxylic acid cycle intermediates, such as succinate and fumarate, metabolite that induces TI by itself (158). In addition, the glycolysis induction favors the generation of NADPH which is an important co-factor for epigenetic enzymes (159), linking the metabolic rewiring with the long-lasting epigenetic imprinting responsible of TI.

### Updates in Trained Immunity-Related Signaling Downstream Dectin-1

In an attempt to place the above-mentioned AKT/mTOR/HIF-1α axis downstream Dectin-1, the implication of the proximal adaptors Syk and Raf-1 was addressed by means of specific inhibitors. These experiments demonstrated that the induction of TI depends on Raf-1 (119, 120). This means that the signaling pathway leading to the trained phenotype does not rely on Syk, raising the question about the role of phagocytosis in the TI induction as a prototypical Syk-independent process downstream Dectin-1. Of note, β-glucan peptide, a soluble derivate of particulate fungal β-glucan has been used to induce TI (160), suggesting that phagocytosis is not required for the induction of the trained process. Nonetheless, this issue demands an experimental clarification.

Elaborating on the discussion about signaling downstream Dectin-1 involved in TI, the expression of long noncoding RNAs (lnRNA) in response to β-glucan has been described as a critical step for the establishment of epigenetic modifications in promoters of proinflammatory genes (161), molecular process that drives the induction of TI (162). In these experiments, the expression of the lnRNA was analyzed in response to β-glucan and TNF-α, showing a dependency on NFAT. However, the seminal work addressing the relevance of lnRNA in TI only analyzed short-term responses following β-glucan stimulation (161). Therefore, the study of lnRNA and NFAT on TI prototypical experiments based on heterologous stimuli would help to solidify the implication of these two factors in the trained process.

As indicated before, the activation of the glycolytic metabolism downstream Dectin-1 is fundamental for the induction of TI (158). In this sense, it is worth to consider that the glucose metabolism is directly induced in response to *Candida albicans* (163). Therefore, for the study of the glycolytic reprogramming during TI, results obtained using the systemic candidiasis model should consider the intrinsic impact of the infection on such metabolism. Interestingly, the mitochondrial metabolism has been also involved in the induction of TI by oxidized low density lipoproteins (oxLDL) (164). Whether this is a specific feature of oxLDL-induced TI, and the relevance of oxidative phosphorylation in the generation of the trained process by different inducers such as Dectin-1 ligands, deserves further studies.

Along these lines, in parallel to the induction of the glycolytic metabolism during Dectin-1-mediated TI, a reduction of the capacity of the mitochondrial electron transport chain takes place (157). Once trained cells use glycolysis as the main ATP supplier, mitochondria could be relegated from this function. This leads to an increase in mitochondrial membrane potential that can result in the generation of ROS (165). Additionally, succinate, which is accumulated during TI (158), drives an extensive superoxide formation from mitochondrial complex-I by reverse electron transport, both processes implicated in the generation of ROS (165, 166). Considering that neutrophils from β-glucan-trained mice suppress tumor growth in a ROS-dependent manner (160), it is tempting to speculate that ROS production might play a role in the generation of TI. This hypothesis is supported by the contribution of the antioxidant glutathione metabolism to the induction of trained phenotype (167).

Related to ROS production downstream Dectin-1, we have already reviewed the relevance of the phosphatase SHIP-1 in this process (73). Based on this notion, we described how SHIP-1 regulates the molecular events downstream Dectin-1 implicated in the generation of TI, such as glycolytic metabolism and the establishment of epigenetic traits on pro-inflammatory promoters (168). This work defined the potential regulation of Dectin-1-triggered responses to modulate TI and therefore, the capacity of improving protective responses mediated by the trained process.

Eventually, the long-term effect of these TI-mediated reactions relies on the rewiring of bone marrow progenitors that will give rise to trained mature cells upon a second challenge (152). The generation of this reprogramming in response to β-glucan recognition would imply that it gains access to the bone marrow niche. This fact has been demonstrated in the case of TI induced by intravenous administration of BCG. β-glucan is classically administered through the intraperitoneal route for the induction of TI, but to the best of our knowledge, no studies have found β-glucan particles in the bone marrow after its systemic administration. In addition, the recognition of that β-glucan by progenitor cells would be required. In this sense, the expression of Dectin-1 in hematopoietic stem and progenitor cells (HSPCs) from the bone marrow can be inferred by their differentiation towards a trained phenotype in response to Dectin-1 ligands such as depleted zymosan (169). Furthermore, this response is defective against *Candida albicans* stimulation in Dectin-1-deficient HSPCs (170). Still, indirect effects mediated by IFN-γ and IL-1β in progenitor myeloid cells have been also demonstrated to mediate the activation of glycolysis and epigenetic reprogramming (151, 171), that consequently generate a trained phenotype in response to β-glucans. Therefore, the definition of actual mechanisms implicated in the reprogramming of bone marrow progenitors in response to Dectin-1 ligands is still poorly-defined, in particular under the light of new findings indicating the transmission of the TI phenotype across generations (172).

### Clinical Relevance of Dectin-1-Mediated Trained Immunity

The fine dissection of signaling pathways triggered downstream Dectin-1 would provide molecular candidates as potential therapeutic or prophylactic targets. It is important to highlight the wide potential of Dectin-1-induced TI against infectious agents as diverse as fungi (119), bacteria (173) or parasites (174).

Furthermore, the administration of β-glucans confers protection also against pathologies of viral etiology such as recurrent respiratory tract infections (175, 176). This knowledge regarding the broad protective potential of TI against virus has fueled the research to understand whether TI could represents a new weapon against the COVID-19 pandemic (177). BCG vaccination (178, 179) or the administration of mucosal immunotherapies such as Trained Immunity-based Vaccines (TIbV) (180) have been shown to confer protection against SARS-CoV-2 infection. Along this line, prophylactic treatments based on β-glucans (181) or their incorporation through the diet (182) have been proposed to serve as a defense against COVID-19, but experimental data are still scarce in this topic.

Therefore, protective trained responses could be induced by the administration of β-glucan in these contexts. But as reviewed above, several endogenous Dectin-1 ligands have been described that could potentially induce TI as well. This is known for alternative endogenous stimuli not characterized as Dectin-1 ligands such as oxidized-LDL (183) implicated in atherosclerosis (184) or even feeding habits (185, 186), that might include β-glucans with potential immunomodulatory capabilities (187).

Taking altogether, and considering the potential application of TI-based approaches to fight pathologies such as allergy (188), cancer (160) or autoimmune diseases (189), a deep understanding of Dectin-1-driven molecular events ignited by different ligands, and their relationship with TI, represents a research field of great interest.

## Open Questions

Under the light of an updated signaling profile triggered downstream Dectin-1, it is clear the relevance of depicting these molecular routes in detail. This knowledge would provide tools to harness these signalings against a broad range of pathologies, motivated by the wide diversity of Dectin-1 ligands, and the large number of potential clinical applications. Why Dectin-1-triggered responses are immunogenic or tolerogenic depending on the settings? Do different ligands induce specific signaling pathways? What is the role of co-regulatory molecules such as tetraspanins in these differential responses? Can we use this dichotomy in our favor? Considering TI as a therapeutic or prophylactic intervention of great interest initiated by Dectin-1, how much of those interrelationships apply to the trained process?

Despite the research on Dectin-1 began more than twenty years ago, there is still a vast field of research to fully understand the implication of the signaling triggered downstream its activation. In this review we have tried to update the current knowledge on this signaling, highlighting gaps of potential research interest. We hope that this review helps to motivate the filling of these gaps.

## Author Contributions

CF conceived the manuscript. MB-G produced the figures. PM-M produced the table. PM-M, MB-G, and CF wrote the manuscript. All the authors discussed and agreed on the final manuscript.

## Funding

The laboratory of CF is funded by Instituto de Salud Carlos III through the project CP20/00106 (Co-funded by European Social Fund "Investing in your future"), by IdiPAZ as a recipient of a Dr. Luis Alvarez fellowship and by Inmunotek (PI-4925).

## Conflict of Interest

The authors declare that the research was conducted in the absence of any commercial or financial relationships that could be construed as a potential conflict of interest.

## Publisher’s Note

All claims expressed in this article are solely those of the authors and do not necessarily represent those of their affiliated organizations, or those of the publisher, the editors and the reviewers. Any product that may be evaluated in this article, or claim that may be made by its manufacturer, is not guaranteed or endorsed by the publisher.
